# Development of antidiabetic drugs from benzamide derivatives as glucokinase activator: A computational approach

**DOI:** 10.1016/j.sjbs.2022.01.058

**Published:** 2022-02-04

**Authors:** Amena Ali

**Affiliations:** Department of Pharmaceutical Chemistry, College of Pharmacy, Taif University, P.O. Box 11099, Taif 21944, Saudi Arabia

**Keywords:** Glucokinase activator, Benzamide, 3D-QSAR, Virtual screening, Pharmacophore analysis, Docking

## Abstract

Hyperglycemia is a condition known for the impairment of insulin secretion and is responsible for diabetes mellitus. Various small molecule inhibitors have been discovered as glucokinase activators. Recent studies on benzamide derivatives showed their importance in the treatment of diabetes as glucokinase activator. The present manuscript showed a computation study on benzamide derivatives to help in the production of potent glucokinase activators. In the present study, pharmacophore development, 3D-QSAR, and docking studies were performed on benzamide derivatives to find out the important features required for the development of a potential glucokinase activator. The generated pharmacophore hypothesis ADRR_1 consisted of essential features required for the activity. The resultant statistical data showed high significant values with R^2^ > 0.99; 0.98 for the training set and Q^2^ > 0.52; 0.71 for test set based on atom-based and field-based models, respectively. The potent compound 15b of the series showed a good docking score via binding with different amino acid residues such as (NH…ARG63), (SO_2_…ARG250, THR65), and π-π staking with (phenyl……TYR214). The virtual screening study used 3563 compounds from ZINC database and screened hit compound ZINC08974524, binds with similar amino acids as shown by compound 15b and crystal ligand with docking scores SP (-11.17 kcal/mol) and XP (-8.43 kcal/mol). Compounds were further evaluated by ADME and MMGBSA parameters. Ligands and ZINC hits showed no violation of Lipinski rules. All the screened compounds showed good synthetic accessibility. The present study may be used by researchers for the development of novel benzamide derivatives as glucokinase activator.

## 1 Introduction

Glucokinase is a hexokinase isozyme, consist of 465 amino acids (molecular weight = 50 kD) present in pancreatic β-cells and liver (postprandial state). Glucokinase catalyzes a reaction that involve the transfer of phosphate from ATP to glucose and the generation of glucose 6-phosphate which is the first step in the direction of synthesis of glycogen and glycolysis (Matschinsky, 1996, Antoine et al., 2009). This reaction is also representing the first rate-limiting step in glucose metabolism. Glucokinase activator (GK-A) worked through two different mechanisms known as lowering the blood glucose level in the liver and increasing insulin secretion in pancreatic β-cells. Therefore, it becomes an interesting target in the present scenario to treat diabetes. Various GK-As have been synthesized, some are under clinical studies and showed promising results to lower blood glucose levels in healthy people and type-2 diabetes mellitus (T2-DM) patients. Glucokinase activator is responsible for several side effects such as hypoglycemia and testicular toxicity (American Diabetes Association, 2009, Waring et al., 2011). To eliminate these side effects, frequent dose regimens and dose titration are preferred. There are two different approaches described to treat hypoglycemic state, one is related to the designing of partial activators (Pfefferkorn et al. 2011) and the second approach is to restrict liver-selective glucose activators (Bebernitz et al., 2009, Massa et al., 2011, Pfefferkorn et al., 2012, Park, 2012, Park et al., 2013).

In the direction to treat diabetes, various benzamide derivatives have been developed as GK-As (Matschinsky and Porte, 2010, Mao et al., 2012). Park, *et al.* identified a novel phenylethyl benzamide GK-A (Park, 2012, Park et al., 2013). Furthermore, Park *et al.* synthesized a series of pyrazole benzamide derivatives as GK-A having 3-methylpyridine and 4-phenoxymethyl sulfone groups for the treatment of T2-DM. Grewal *et al.* synthesized a series of benzamide 3,5-disubstituted analogues and evaluated them for GK activation activity. These analogues showed considerable antihyperglycemic activity in the animal models (Grewal et al. 2019). In a study, Charaya *et al.* synthesized thiazole-2-yl benzamide derivatives from benzoic acid and evaluated them for GK activation activity (Charaya et al. 2018). A benzamide derivative PF-04937319 **(1)** is under phase-1 clinical trial for the treatment of diabetes.

**Figure:**
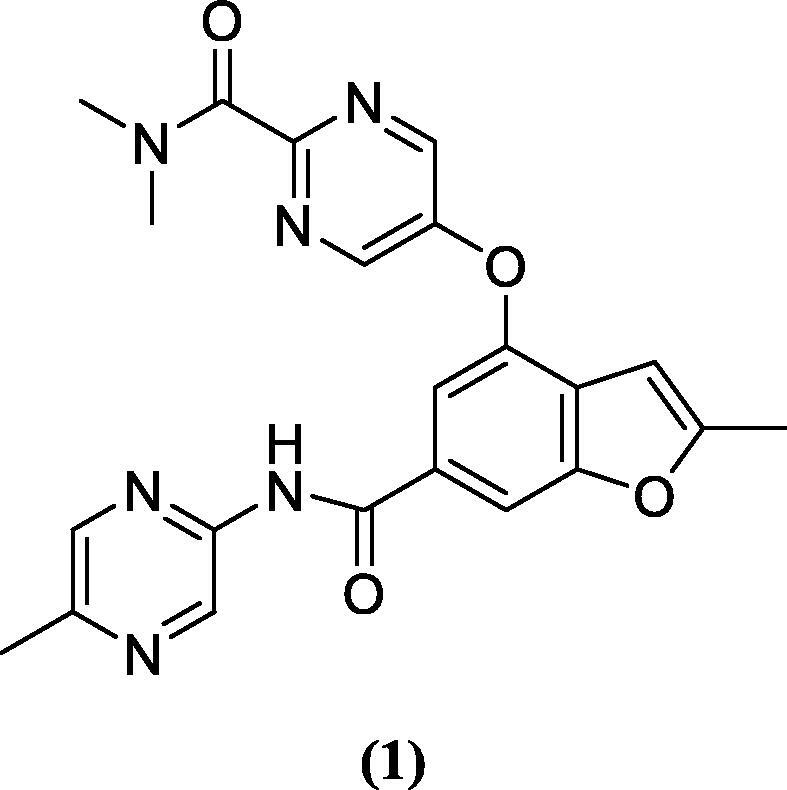

On the basis of previously synthesized compounds (Park, 2012, Park et al., 2013), structure-based drug design was performed with pharmacophore development, 3D QSAR, and docking simulations for the determination of potent GK-A by using the PHASE module (Schrodinger). Pharmacophore development determines the important features required for the activity and can be used for 3D QSAR and virtual screening studies. Field and atom-based 3D-QSAR models were developed for the determination of statistical data results of correlation between molecules and their properties. A virtual screening study on the ZINC database generated the potent compounds as GK-A. Docking study revealed the important interactions with amino acids required for activity. On the other hand, the MMGBSA (Molecular Mechanics Generalized Born Surface Area) method was used to predict the binding free energy of the docked molecules. The current findings of the study may be utilized as a guiding tool for the development of novel and effective GK-A.

## 2 Methodologies

### 2.1 Software

The 3D-QSAR, pharmacophore modeling and docking were performed by Schrodinger module (Park et al., 2014, Park et al., 2015).

### 2.2 Dataset

**Fig. 1:**
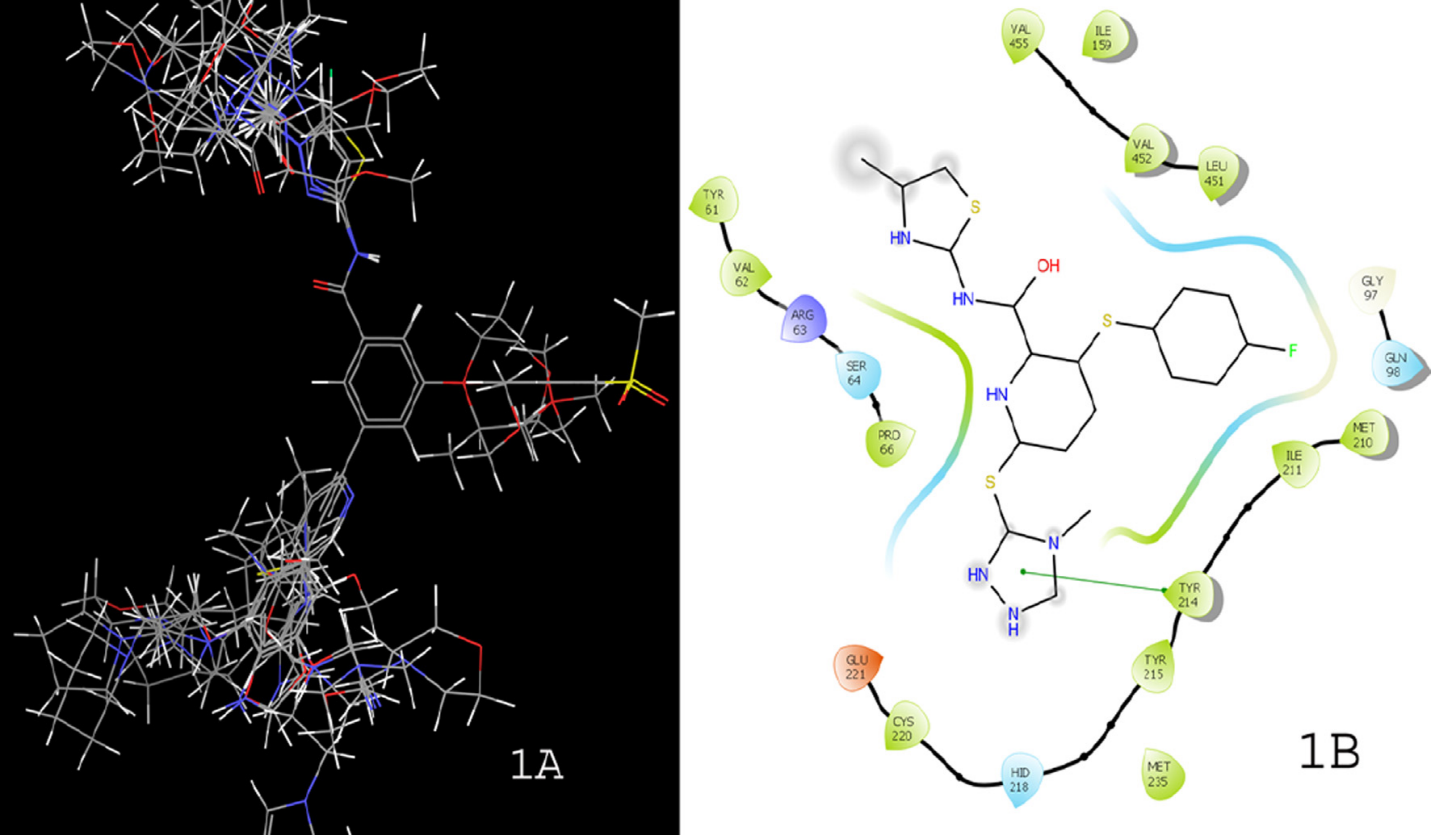
(1A) Common scaffold alignment of 43 benzamide derivatives; (1B) Binding interactions of crystal ligand with protein 3AOI.

A dataset of 43 benzamide derivatives was taken for the development of pharmacophore, 3D-QSAR, virtual screening, and docking studies (Table 1). The IC_50_ values taken from biological activities were converted into pIC_50._ Data set were divided into active for ‘higher activity’ and inactive for ‘lower activity’ compounds and the remaining were counted in the category of intermediate. The alignment on common scaffold of 43 benzamide derivatives is given in Fig. 1A**.** The PDB ID-3A0I was used for the docking studies. The crystal ligand of this protein used for comparison of docking outcomes are presented in Fig. 1B (Andreoli et al. 2014).

**Table 1:** In vitro IC_50_ and PIC_50_ values for GK activity of pyrazole benzamide derivatives (Park, 2012, Park et al., 2013).

**15a-18k**			**19a-19e**					**13a1-22e1**	
**C**	**R1**	**R2**	**AP-A (IC_50_, µM)**	**AP-A (pIC_50_)**	**C**	**R1**	**R2**	**AP-A (IC_50_, µM)**	**AP-A (pIC_50_)**
**16a**	H	H	0.095	7.02	**16b1**		–	0.301	6.52
**16b**	H	F	0.238	6.62	**16c1**		–	0.025	7.60
**15a**		H	0.103	6.99	**18a1**		–	0.021	7.68
**15b**		H	0.005	8.30	**18b1**		–	0.012	7.92
**18a**		H	50	4.30	**18c1**		–	0.03	7.52
**18b**		H	0.212	6.67	**18d1**		–	0.034	7.47
**18c**		H	0.688	6.16	**19**		–	0.027	7.57
**18d**		H	2.43	5.61	**21**		–	0.039	7.41
**18e**		H	7.46	5.13	**20a1**		–	0.037	7.43
**18f**		H	50	4.30	**20b1**		–	0.163	6.79
**18 g**		H	0.008	8.10	**20c1**		–	0.006	8.22
**18 h**		H	0.033	7.48	**20d1**		–	0.006	8.22
**18i**		H	0.463	6.33	**20e1**		–	0.006	8.22
**18j**		H	0.106	6.97	**20f1**		–	0.02	7.70
**18 k**		H	50	4.30	**20 g1**		–	0.039	7.41
**19a**	CH_3_	–	0.807	6.09	**20 h1**		–	0.009	8.05
**19b**		–	50	4.30	**22a1**		–	0.053	7.28
**19d**		–	0.441	6.36	**22b1**		–	0.046	7.34
**19e**		–	0.315	6.50	**22c1**		–	0.006	8.22
**13a1**		–	1.87	5.73	**22d1**		–	0.007	8.15
**13b1**		–	0.006	8.22	**22e1**		–	0.016	7.80
**16a1**		–	7.7	5.11					

C: Compound; AP-A: Antiproliferative activity.

### 2.3 Preparation of ligands

The molecular structure of benzamide derivatives was converted from 2D to 3D by using LigPrep of Schrodinger software. The OPLS_2005 force field was taken for the preparation of ligands. Further, the prepared molecules were taken for 3D-QSAR and docking simulations (Schrodinger, 2017, Ali and Ali, 2021).

### 2.4 Pharmacophore development

The pharmacophore model was developed by using PHASE module, where all 43 ligands were aligned on common scaffold and generated conformers using the macromodel search method (Ali and Ali 2021). PHASE module consists six different pharmacophore features including hydrogen bond acceptor (HBA), hydrogen bond donor (HBD), aromatic ring, hydrophobic group, positive ionizable, and negative ionizable groups (Rajeswari et al. 2014). The maximum number of sites was fixed to 5 which further responsible for the generation of the topmost 20 different hypotheses. For the generation of these hypotheses, ligands were categorized into active, inactive, and intermediate.

**Fig. 2:**
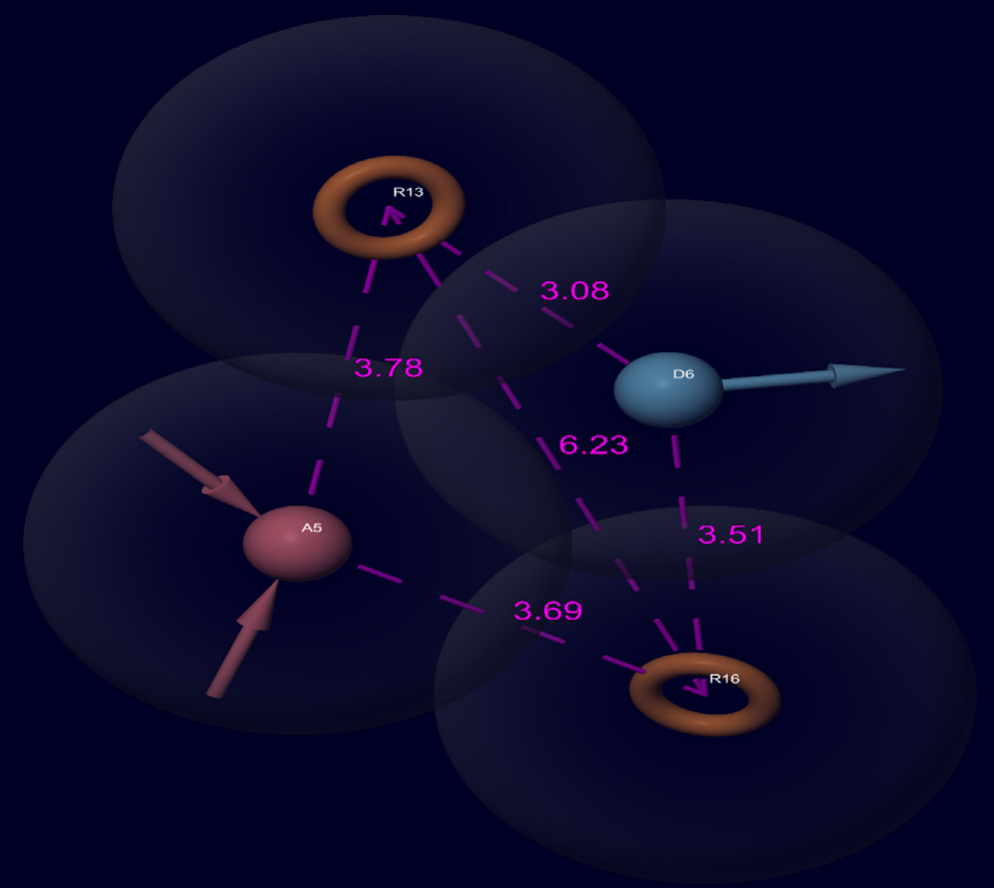
Pharmacophore model (ADRR_1). Model represents various features by arrow like acceptor (A; pink-colour), donor (D: grey colour), hydrophobic and aromatic ring (R: brown colour) features.

The active ligand was set as for those which had pIC_50_ value of more than 8 whereas inactive ligands considered for those had pIC_50_ value less than 6.6, except these all-other ligands were set as intermediates. The final data consisted of 9 actives and 14 inactive ligands. The hypothesis was generated by using 9 actives and 1 Å box size and 2 Å site distance. The all-generated hypotheses were ranked based on different scores depicted in Table 2 (Crisan et al., 2019, Sakkiah et al., 2014). The hypothesis ADRR_1 (Fig. 2) showed the top scoring features with one HBA, one HBD, and two aromatic rings. The hypothesis determines the essential features required for the binding with receptor for a particular activity. A total of 20 pharmacophore hypotheses were developed which were ranks according to the score hypothesis (Table 2).

**Table 2:** The generated pharmacophore hypotheses.

**N.**	**Hypothesis**	**Phasehypo-S**	**Survival-S**	**Inactive-S**	**Site-S**	**Vector-S**	**Volume-S**	**Selectivity-S**
1	DHHRR_1	1.25	5.061	1.433	0.689	0.927	0.537	2.063
2	ADRR_1	1.18	5.032	1.234	0.982	0.922	0.409	1.955
3	AHHRR_1	1.17	4.964	1.283	0.725	0.957	0.502	1.936
4	AAHHR_1	1.16	4.829	1.329	0.772	0.957	0.521	1.733
5	ADHRR_1	1.15	4.813	1.698	0.775	0.898	0.411	1.775
6	ADHHR_1	1.14	4.805	1.237	0.696	0.926	0.544	1.793
7	ADHHR_2	1.13	4.798	1.269	0.701	0.983	0.465	1.804
8	AAHHR_2	1.11	4.777	1.333	0.607	0.937	0.525	1.864
9	ADHHR_3	1.10	4.764	1.464	0.671	0.961	0.491	1.797
10	AAHRR_1	1.09	4.719	1.298	0.854	0.724	0.481	1.883
11	AADHR_1	1.09	4.713	1.778	0.985	0.951	0.480	1.451
12	HHRR_1	1.09	4.572	1.391	0.742	0.923	0.554	1.509
13	AHHR_1	1.08	4.531	1.129	0.873	0.905	0.545	1.363
14	DHRR_1	1.07	4.524	1.645	0.824	0.846	0.410	1.489
15	AHHR_2	1.07	4.508	1.616	0.740	0.900	0.537	1.485
16	DHHR_1	1.06	4.469	1.513	0.668	0.944	0.548	1.465
17	ADHR_1	1.06	4.459	1.548	0.996	0.928	0.467	1.222
18	AAHR_1	1.06	4.426	1.766	0.995	0.937	0.465	1.183
19	AHRR_1	1.00	4.394	1.683	0.762	0.886	0.409	1.383
20	AHRR_2	0.96	4.389	1.386	0.923	0.695	0.498	1.495

S: Scores

### 2.5 3D-QSAR

**Fig. 3:**
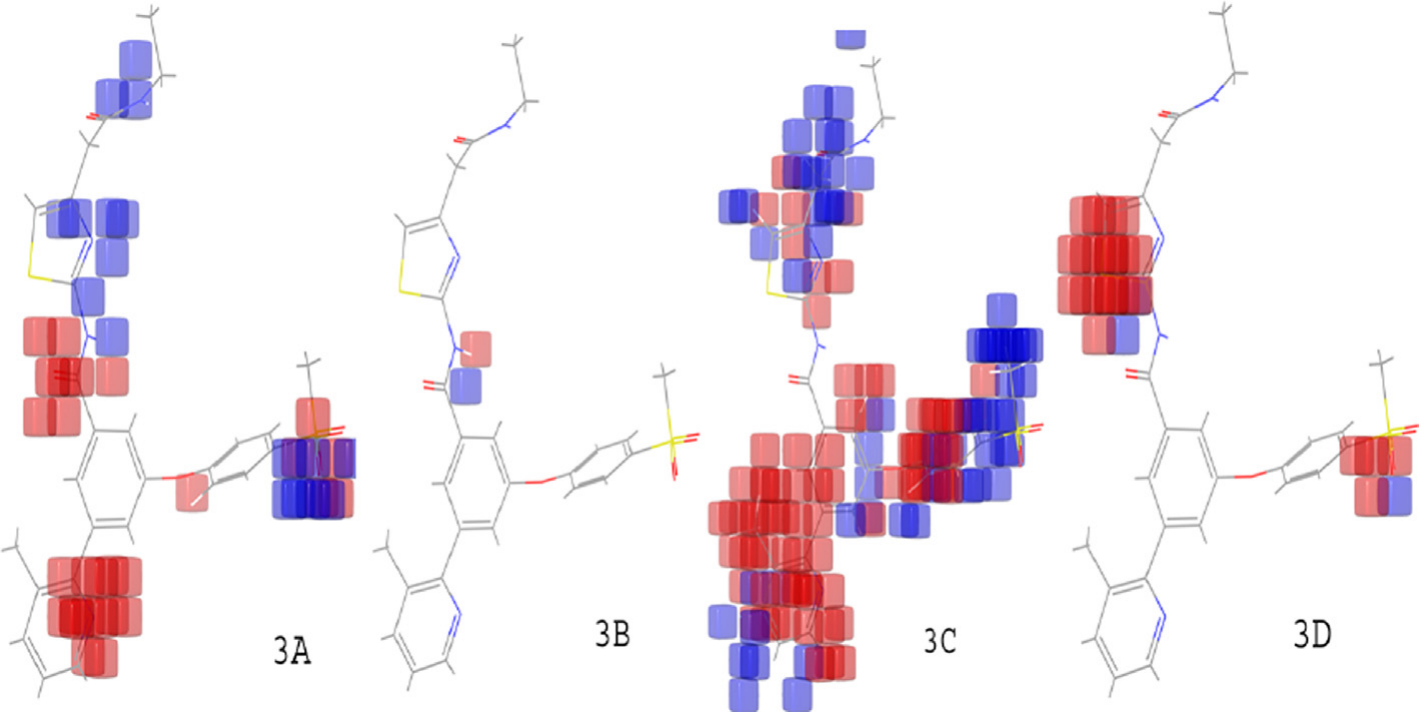
3D contour maps, atom-based: (3A) EWG; (3B) HBD group; (3C) Hydrophobic group; (3D) Positive ionic group.

**Fig. 4:**
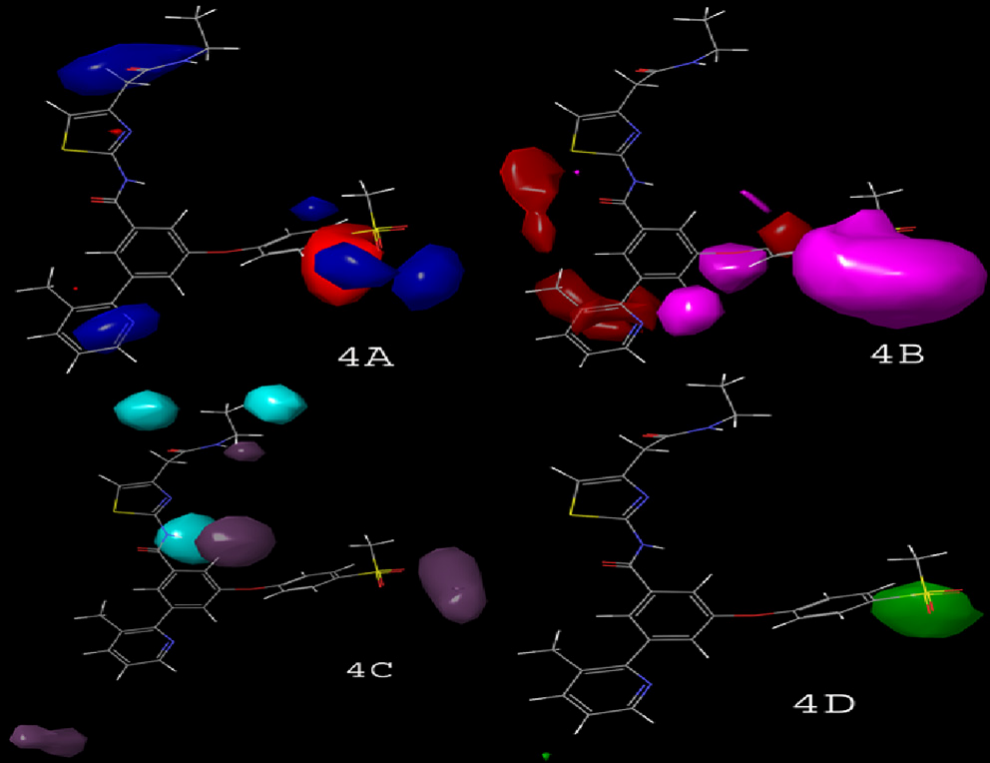
Different field contour maps: (4A) Electrostatic (blue {favored}, red {disfavored}); (4B) HBA (red {favored}, magenta {disfavored}); (4C) HBD (purple {favored}, cyan {disfavored}); (4D) Steric (green {favored}, yellow {disfavored}).

3D-QSAR models were developed by two techniques known as field-based and atom-based QSAR. The developed models were showed the essential parameters required for activity by correlating the structure features with biological activity. All 43 benzamide derivatives were separated into a training set with 75% and test set with 25% compounds using 4 PLS factors (Kar et al. 2010). Best models were generated by QSAR models (atom and field-based) described in Table 3. Statistics for atom-type fraction and field type fraction were summarized in Table 4, Table 5. In the atom-based model, 35 molecules were selected for the training, and 8 molecules for the test set, whereas in field-based 31 and 12 molecules were taken for the training and the test set, respectively (Table 6). The contour maps generated by atom-based and field-based models are presented in Fig. 3, Fig. 4, respectively. These maps are represented as hydrophobic, steric, donor, acceptor, and electrostatic fields (Peng et al. 2017).

**Table 3:** Statistical data generated by atom based (A.B.) and field based (F.B.) models.

**P.F.**	**R^2^**		**R^2^ CV**		**Q^2^**		**SD**		**RMSE**		**Stability**		**P-r**		**F**	
	**A.B.**	**F.B.**	**A.B.**	**F.B.**	**A.B.**	**F.B.**	**A.B.**	**F.B.**	**A.B.**	**F.B.**	**A.B.**	**F.B.**	**A.B.**	**F.B.**	**A.B.**	**F.B.**
1	0.80	0.63	0.59	0.40	0.41	0.72	0.59	0.55	1.06	0.65	0.89	0.91	0.67	0.88	81.90	41.30
2	0.95	0.86	0.57	0.54	0.45	0.61	0.57	0.56	1.02	0.77	0.63	0.77	0.75	0.82	195.20	71.20
3	0.98	0.94	0.59	0.61	0.50	0.69	0.59	0.31	0.97	0.69	0.63	0.72	0.80	0.87	251.20	124.50
4	0.99	0.98	0.62	0.64	0.52	0.71	0.62	0.19	0.96	0.66	0.65	0.70	0.82	0.86	299.80	271.90

P.F.: PLS factor; A.B.: Atom based; F.B.: Field based

**Table 4:** Statistical fraction data procured from atom based model.

**Factors**	**Hydrogen donor**	**Hydrophobic group**	**Positive group**	**Electron withdrawing group**	**Other**
1	0.013	0.675	0.247	0.065	0.013
2	0.012	0.700	0.243	0.044	0.012
3	0.012	0.703	0.241	0.045	0.012

**Table 5:** Statistical fraction data procured from field based model.

**Factors**	**Steric**	**Electrostatic**	**Hydrophobic**	**Hydrogen Acceptor**	**Hydrogen Donor**
1	0.361	0.101	0.242	0.226	0.07
2	0.411	0.098	0.262	0.151	0.077
3	0.403	0.099	0.276	0.138	0.085

**Table 6:** The IC_50_ value (Actual vs predicted) generated by atom-based and field-based 3D-QSAR model using PLS factor 4.

**N.**	**Ligand**	**QSAR set (A.B.)**	**Observed activity**	**Predicted activity (A.B)**	**QSAR set** **(F.B)**	**Predicted activity (F.B)**
1	15b	Training	8.301	6.70	Training	8.49
2	13b1	Training	8.222	6.91	Training	8.18
3	20c1	Training	8.222	7.50	Training	8.32
4	20d1	Training	8.222	8.16	Training	8.17
5	20E_1	Test	8.222	8.16	Test	7.88
6	22c1	Training	8.222	7.29	Test	7.26
7	22d1	Training	8.155	8.12	Training	8.16
8	18g	Test	8.097	7.92	Training	8.01
9	20h1	Test	8.046	7.17	Test	7.47
10	18b1	Training	7.921	7.76	Training	8.05
11	22E_1	Training	7.796	7.85	Training	7.60
12	20f1	Training	7.699	7.50	Training	7.88
13	18a1	Training	7.678	7.69	Training	7.73
14	16c1	Training	7.602	7.29	Training	7.29
15	19	Training	7.569	7.60	Test	7.76
16	18c1	Training	7.523	7.66	Test	7.43
17	18h	Training	7.481	7.25	Training	7.32
18	18d1	Test	7.468	7.35	Training	7.62
19	20a1	Training	7.432	7.52	Training	7.53
20	20g1	Training	7.409	7.42	Test	7.16
21	21	Training	7.409	7.43	Training	7.23
22	22b1	Training	7.337	7.30	Training	7.16
23	22a1	Training	7.276	7.28	Training	7.41
24	16a	Training	7.02	6.89	Training	6.92
25	15a	Test	6.987	6.87	Training	6.69
26	18j	Training	6.975	6.34	Test	6.62
27	20b1	Training	6.788	6.64	Training	6.99
28	18b	Training	6.674	6.76	Test	6.24
29	16b	Training	6.62	6.38	Training	6.23
30	16b1	Training	6.521	6.35	Training	6.18
31	19e	Test	6.502	6.60	Training	6.48
32	19d	Training	6.356	6.27	Training	6.31
33	18i	Training	6.334	6.32	Training	6.33
34	18c	Test	6.162	6.25	Test	5.16
35	19a	Training	6.093	6.29	Training	5.99
36	13a1	Training	5.728	6.01	Training	5.86
37	18d	Training	5.614	5.57	Test	6.09
38	18e	Training	5.127	5.03	Test	4.65
39	16a1	Training	5.114	6.72	Training	5.23
40	18a	Training	4.301	4.18	Test	5.70
41	18f	Training	4.301	5.47	Training	4.45
42	18k	Test	4.301	5.69	Training	4.37
43	19b	Training	4.301	5.23	Training	4.44

A.B.: Atom based; F.B.: Field based

### 2.6 Target protein prediction

The potent compounds of the series were selected and submitted to the Swiss-Target-Prediction tool. The said tool predicted various protein targets, in which GK-A was the most appropriate target for the selected molecules.

### 2.7 Docking analysis

The PDB ID-3A0I consists a three-dimensional structure of GK-A and was downloaded through protein data bank (Tagami et al. 2010). The docking study was performed to determine the binding scores between ligand and receptor by using the Glide module. The SP and XP methodologies were used for all 43 ligands with PDB ID-3A0I (Table 7). The MMGBSA based rescoring technique was used for the prediction of binding free energy calculation between ligands and receptor molecule (Table 8). However, DFT studies can give more accurate assessment of binding/docking (Van Mourik et al., 2014).

**Table 7:** Docking scores generated through the Glide module.

**N.**	**Ligand**	**D.S. (XP)** **kcal/mol**	**D.S. (SP)** **kcal/mol**	**N.**	**Ligand**	**DS (XP)** **kcal/mol**	**DS (SP)** **kcal/mol**
1	18d	−8.854	−12.715	23	19d	−10.014	−9.62
2	19b	−10.686	−12.412	24	13a1	−11.856	−9.56
3	19e	−11.07	−11.731	25	13b1	−10.242	−9.519
4	19a	−10.768	−11.467	26	18b	−10.21	−9.396
5	16b	−9.207	−11.361	27	18a	−9.761	−9.059
6	16a	−9.261	−11.296	28	19	−8.507	−9.03
7	15a	−9.351	−11.284	29	18b1	−8.284	−9.001
8	18k	−9.258	−11.149	30	18d1	−9.406	−8.769
9	15b	−9.047	−11.088	31	20d1	−9.615	−8.707
10	18h	−8.742	−10.866	32	22b1	−8.48	−8.701
11	18c	−8.522	−10.622	33	20h1	−11.053	−8.694
12	18e	−7.234	−10.615	34	16b1	−8.827	−8.653
13	20E_1	−8.191	−10.613	35	20c1	−8.548	−8.653
14	18f	−9.513	−10.536	36	20g1	−8.464	−8.57
15	18g	−11.172	−10.443	37	22a1	−7.801	−8.548
16	18j	−7.131	−10.427	38	16a1	−12.379	−8.513
17	18i	−9.313	−10.184	39	22E_1	−9.187	−8.274
18	18a1	−10.037	−9.95	40	22c1	−7.757	−7.804
19	20a1	−8.082	−9.897	41	20b1	−5.211	−7.388
20	18c1	−11.886	−9.767	42	20f1	−10.515	−6.959
21	16c1	−10.364	−9.703	43	22d1	−7.929	−6.476
22	21	−8.751	−9.679				

DS: Docking score; Extra precision: XP; SP: Standard precision.

**Table 8:** The scores calculated by different docking methodologies used in the present study with their rescoring values calculated by MMGBSA method.

**C**	**PDB ID: 3AOI**			
	**D.S. (XP)** **kcal/mol**	**D.S. (SP)** **kcal/mol**	**D.S. (HTVS)** **kcal/mol**	**dG bind** **kcal/mol**
18g	−11.172	−10.443	−10.65	−81.31
13b1	−10.242	−9.519	−7.85	−74.62
19e	−11.07	−11.73	−6.38	−83.57
19a	−10.77	−11.47	−9.26	−80.57
16b	−9.21	−11.36	−10.78	−84.33
16a	−9.26	−11.30	−11.47	−81.96
15b	−9.05	−11.09	−9.24	−89.31
ARRY-403	−10.259	−10.101	−9.26	−72.62
RO-5305552	−6.707	−9.074	−7.75	−39.15
Piragliatin	−8.345	−8.144	−7.24	−39.35
Glucokinase activator 1	−3.36	−5.782	−8.37	−0.96
ZINC08974524	−8.43	−11.17	−10.26	−19.55
ZINC00656909	−8.715	−10.887	−8.62	−56.96
ZINC31812808	−8.40	−8.223	−9.37	−76.4
ZINC14791611	−8.294	−10.879	−8.28	−29.58
ZINC05204145	−7.984	−7.323	−8.32	−53.31
ZINC01114130	−7.854	−10.21	−9.65	−37.62
ZINC09712705	−7.764	−8.962	−9.78	−47.96
ZINC14462664	−7.65	−10.997	−7.47	−75.4
ZINC02474054	−6.186	−4.208	−6.25	−8158

D.S.: Docking score; XP: Extra precision; SP: Standard precision; HTVS: High-throughput virtual screening.

### 2.8 ADME prediction studies

The top scored compounds were analyzed by different ADME properties such as drug-likeness, solubility, and pharmacokinetic studies. The QikProp software was used to calculate ADME properties (Table 9). Further, the Swiss-ADME tool was used to evaluate additional parameters like cytochrome profile of the drug with permeation through different other barriers.

**Table 9:** ADME predictions of top scored compounds.

**C**	**QP log Po/w^1^**	**QPP- Caco^2^**	**QP log B.B.^3^**	**QPP-MDCK^4^**	**QP log Khsa^5^**	**MetR^6^**	**PHOA^7^**
18g	3.524	62.806	−1.768	46.094	0.214	6	66.803
13b1	3.606	2683.955	−0.698	1438.153	−0.05	3	100
19e	3.411	290.144	−2.14	130.171	−0.097	4	78.033
19a	3.746	323.686	−1.674	146.509	0.346	2	93.806
16b	3.784	251.098	−1.595	332.05	0.266	3	92.054
16a	3.544	300.192	−1.505	262.463	0.187	3	92.039
15b	3.472	76.153	−1.903	56.786	−0.114	7	67.996
ARRY-403	2.664	170.959	−1.879	200.448	−0.156	6	82.508
GK-A 1	4.515	22.249	−2.361	47.821	0.115	3	64.54
Piragliatin	3.048	435.091	−1.217	374.987	0.038	7	92.016
RO-5305552	2.775	199.82	−2.015	174.056	−0.177	4	84.373
ZINC08974524	4.32	2282.231	−0.392	2329.878	0.362	4	100

Predicted [1: Octanol/water partition coefficient; 2: Caco-2 cell permeability (nm/s); 3: Brain/blood partition coefficient; 4: Apparent MDCK cell permeability (nm/s); 5: Human serum albumin binding]; 6: Number of metabolic reactions; 7: Percent human oral absorption.

## 3 Results

### 3.1 Analysis of pharmacophore modeling

**Fig. 5:**
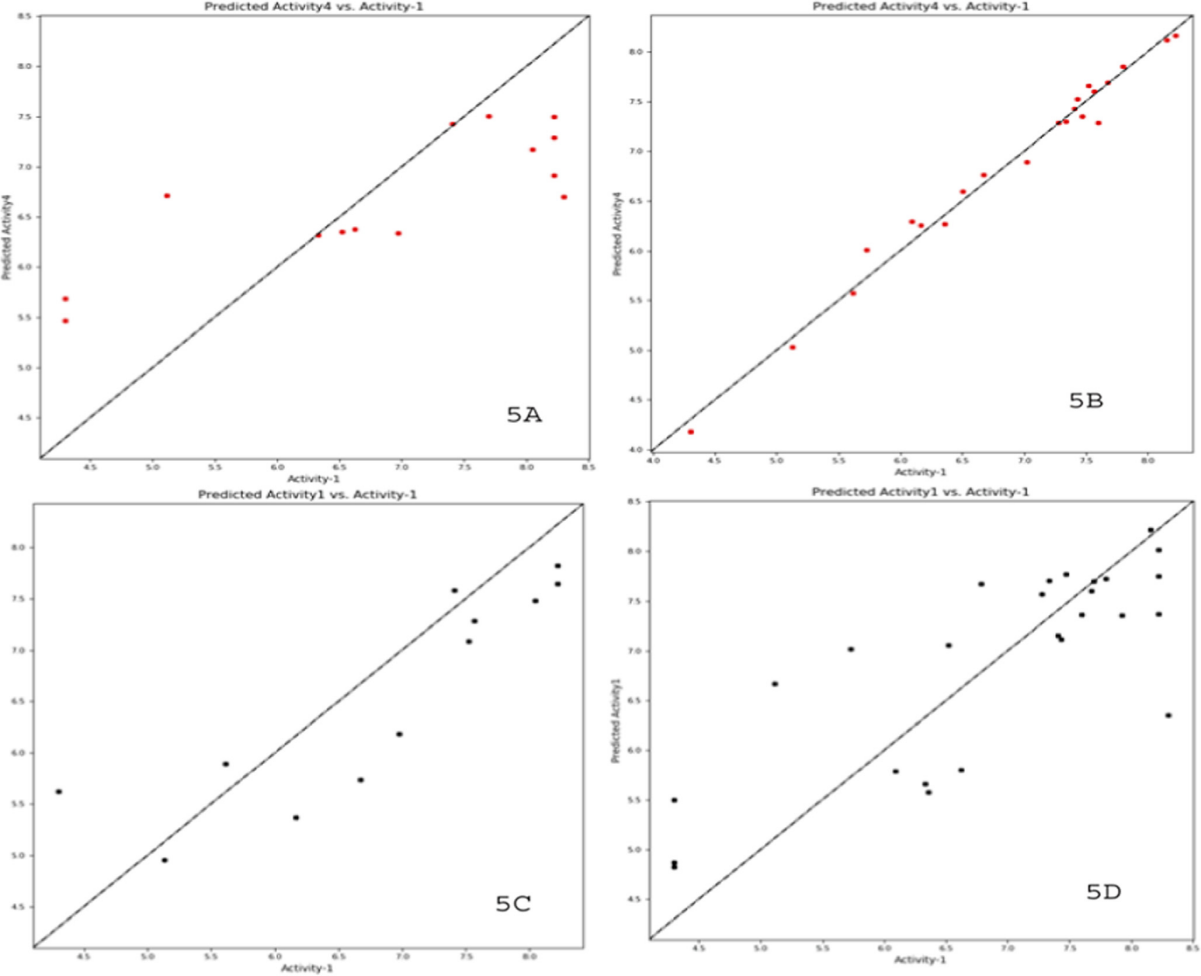
Correlation between test (5 A & C) and training (5B & D) set compounds by using atom and field-based 3D QSAR models.

The generated pharmacophore hypotheses with different scores are presented in Table 2. The hypothesis ADRR_1 was chosen as the best hypothesis with phase hypo-score = 1.18, survival score = 5.03, and site score = 0.98. The field-based and atom-based QSAR studies showed reliable statistical parameters with different evaluation factors. The results showed internal validation parameters such as R^2^ values 0.99, 0.98; R^2^CV values 0.62, 0.64; Q^2^ values 0.52, 0.71; SD values 0.62, 0.19; RMSE values 0.96, 0.66 and F values 299, 271 for atom-based and field-based models, respectively (Table 3). The scatter plots for both models are shown in Fig. 5.

### 3.2 3D-QSAR

The contour maps showed the correlation between different bioactivities by various substituents on the core moiety (Fig. 3). The correlation between actual and predicted activities of test and training-set compounds for atom-based QSAR is depicted in Fig. 5. The contour maps generated by field-based model include HBA, HBD, steric, electrostatic, and hydrophobic fields (Fig. 4). Different substituent groups on the potent compound (16a) described by various colour responsible for increase or decrease in activity. The correlation between actual and predicted activities of test and training-set compounds for field-based QSAR is depicted in Fig. 5.

### 3.3 Docking and virtual screening studies

**Fig. 6:**
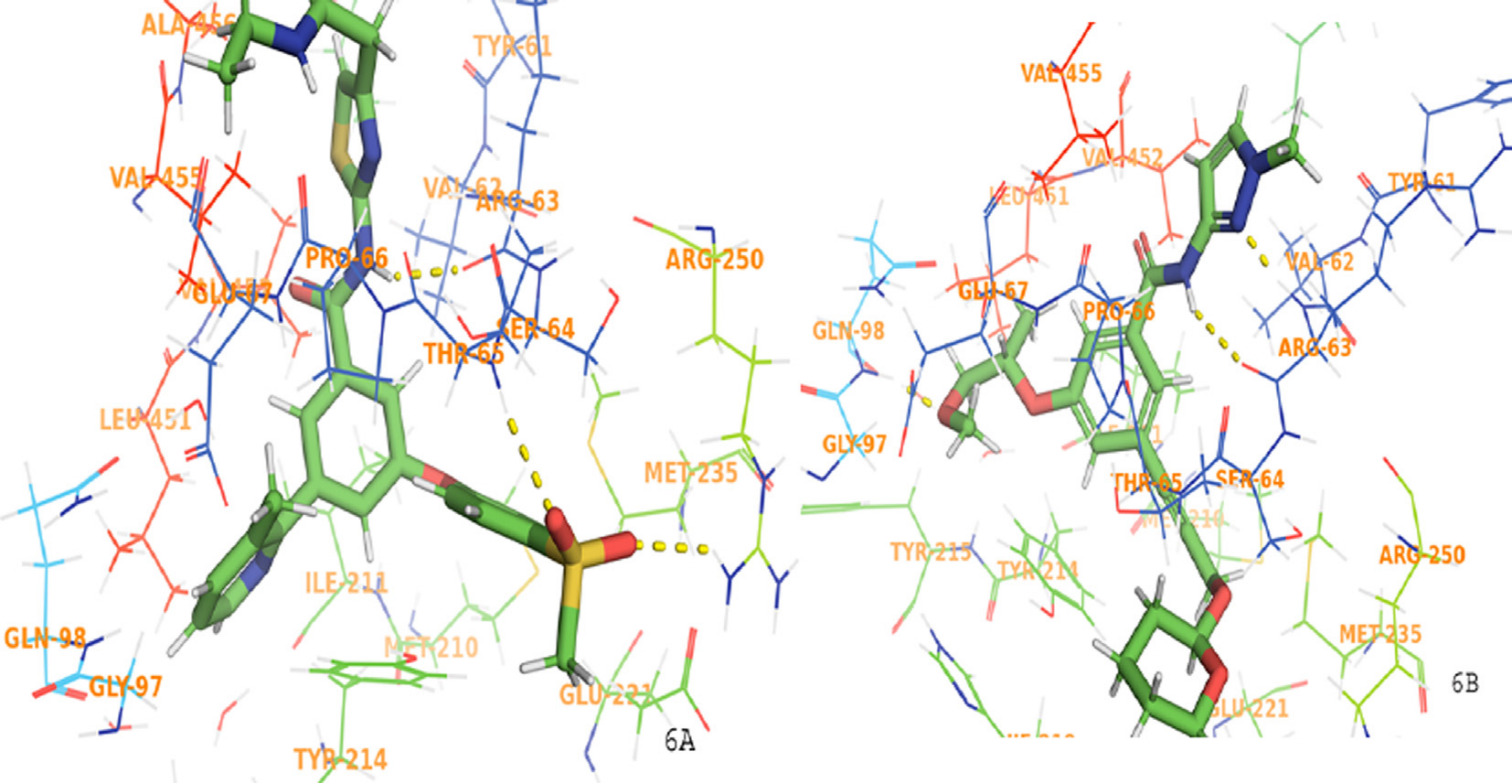
Ligand interaction diagram for higher scoring compound 15b (6A) and 18 g (6B) in docking study.

**Fig. 7:**
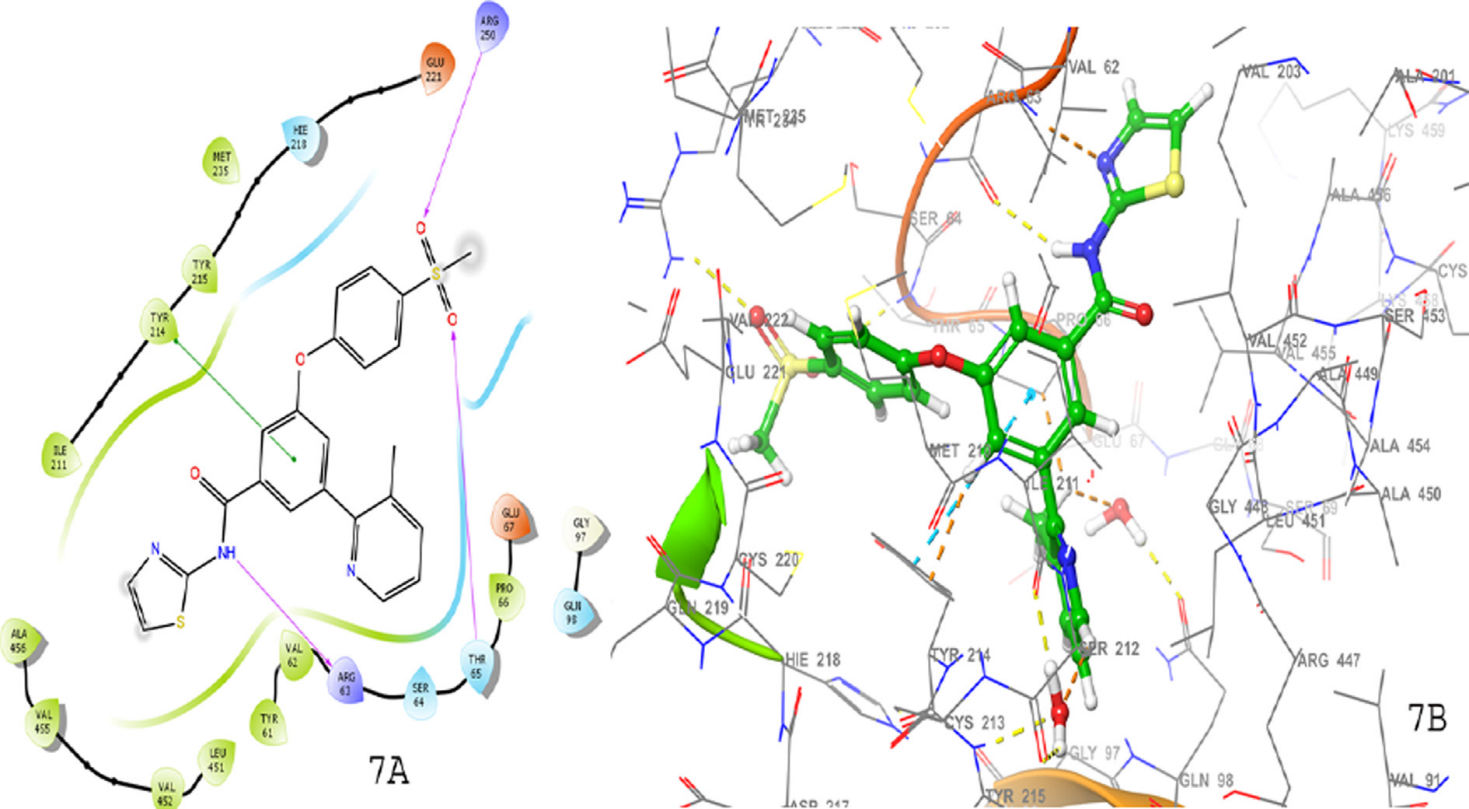
(7A) 2D and (7B) 3D docking interactions represented by compound 16a.

**Fig. 8:**
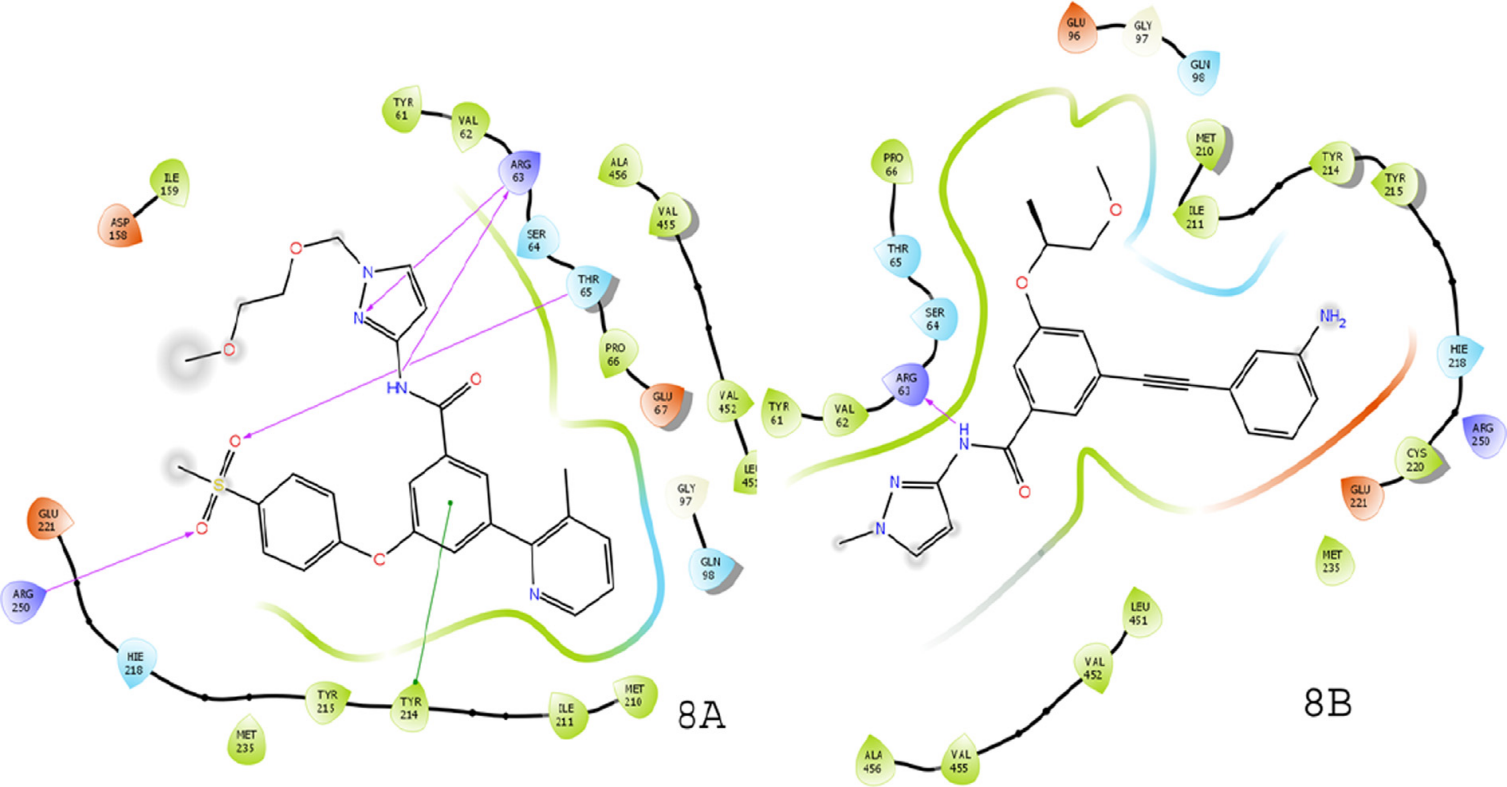
Docking interactions represented by compound 19e (8A) and 19 (8B).

The PDB ID-3A0I was taken for docking purposes to evaluate binding interactions of potent ligands and ZINC compounds. The docking scores were compared with the observed activity. Compounds, 18g, 13b1, 19e, 19a, 16b, 16a, and 15b showed binding interactions with important amino acids required for GK-A. The amino acids bind with both compounds 15b and 18g were ARG250 and THR65, ARG63 as shown in Fig. 6. Compound 16a also showed good binding interactions described in Fig. 7. The docking score of the potent compounds of the series compared with standard drugs such as glucokinase activator 1, piragliatin, ARRY-403**,** and RO-5305552 are described in Table 8. The binding interactions of other potent compounds 19e and 19 are displayed in Fig. 8.

### 3.4 MMGBSA-based rescoring

The rescoring of docked structure of ligand and protein was performed by the MMGBSA-based method (molecular mechanics energies combined with the Poisson–Boltzmann or generalized Born and surface area continuum solvation). The screened ZINC hit compound ZINC08974524 (complex of ZINC08974524: 3A0I) showed docking score of −8.428 kcal/mol. Other ZINC hits such as ZINC00656909, ZINC31812808, ZINC14791611, ZINC05204145, ZINC01114130, ZINC09712705 and ZINC14462664 showed XP docking scores −8.715, −8.40, −8.294, −7.984, −7.854, −7.764 and −7.65 kcal/mol, respectively. All ZINC compounds showed negative value of dG binding energies as compared with standards (Table 8).

### 3.5 ADME properties calculation

ADME properties were calculated by the QikProp module, comprise of one another SwissADME tool. These parameters were within the acceptable range for ligands and ZINC hits (Table 9). The compounds of the series showed drug-likeness properties with no violation of the Lipinski rule. The bioavailability score of compounds showed the value of 0.55.

## 4 Discussion

The final hypothesis consisted of one HBD, one HBA, and two aromatic ring structures. The hypothesis **(**ADRR_1**)** showed alignment with other molecules of the series and displayed good correlation between structure and bioactivity. The features of the hypothesis were further taken for screening of ZINC compounds from the ZINCPHARMER (http://zincpharmer.csb.pitt.edu/) online tool. The atom based QSAR maps indicated the influence on bioactivity by the addition of substituents on the nucleus. The blue contour maps showed an increase in activity, whereas red maps showed decrease in activity. The compound 16a showed alignment with pharmacophore hypothesis ADRR_1 with different colours on their substituents. The electron-withdrawing group (EWG) substitution on benzamide derivatives exhibited increase in the activity, whereas EWG substitution on phenyl ring showed decrease in activity, as represented by red maps (Fig. 3). The HBD group addition showed no changes in activity. The hydrophobic group addition connected to the amide group displayed increase in activity, whereas the introduction of such group at phenyl ring decreased the potency and showed mixed activity throughout the ring. However, the addition of positive and negative ionic groups showed decrease in the activity.

In field based QSAR contour map electrostatic group contains the blue colour at the amide group linked heterocyclic compounds that showed the introduction of electron positive group at the site responsible for increase in the activity. The phenyl ring connected to heterocyclic ring with electron positive group may decrease or increase the activity. The HBA group introduction at phenyl ring connected group may increase the activity. The addition of HBD group at the amide group may increase the activity. The group contains steric field with green color in phenyl ring substituted group may be responsible for increase in activity. The potent compound 15b of the series showed good docking score using interactions with amino acid residues (NH…ARG63), (SO_2_…ARG250, THR65), and π-π staking with (phenyl……TYR214). Compound 16a also showed good binding interactions with amino acid residues such as TRY214, ARG250, THR65, and ARG63 (docking score −11.296 kcal/mol), important for GK-A activity (Fig. 7). The docking scores and the amino acid residues of the potent compounds of the series compared with standard drugs such as glucokinase activator 1 (−3.36 kcal/mol), piragliatin **(**−8.345 kcal/mol**)**, ARRY-403 **(**−10.259 kcal/mol**),** and RO-5305552 **(**−6.707 kcal/mol**)** (Table 8). Furthermore, the other potent compounds 19e and 19 from both the series showed good binding interactions with THR65, ARG63, ARG250, and TYR214 essential amino acids that are important for a GK-A drug (Fig. 8).

**Fig. 9:**
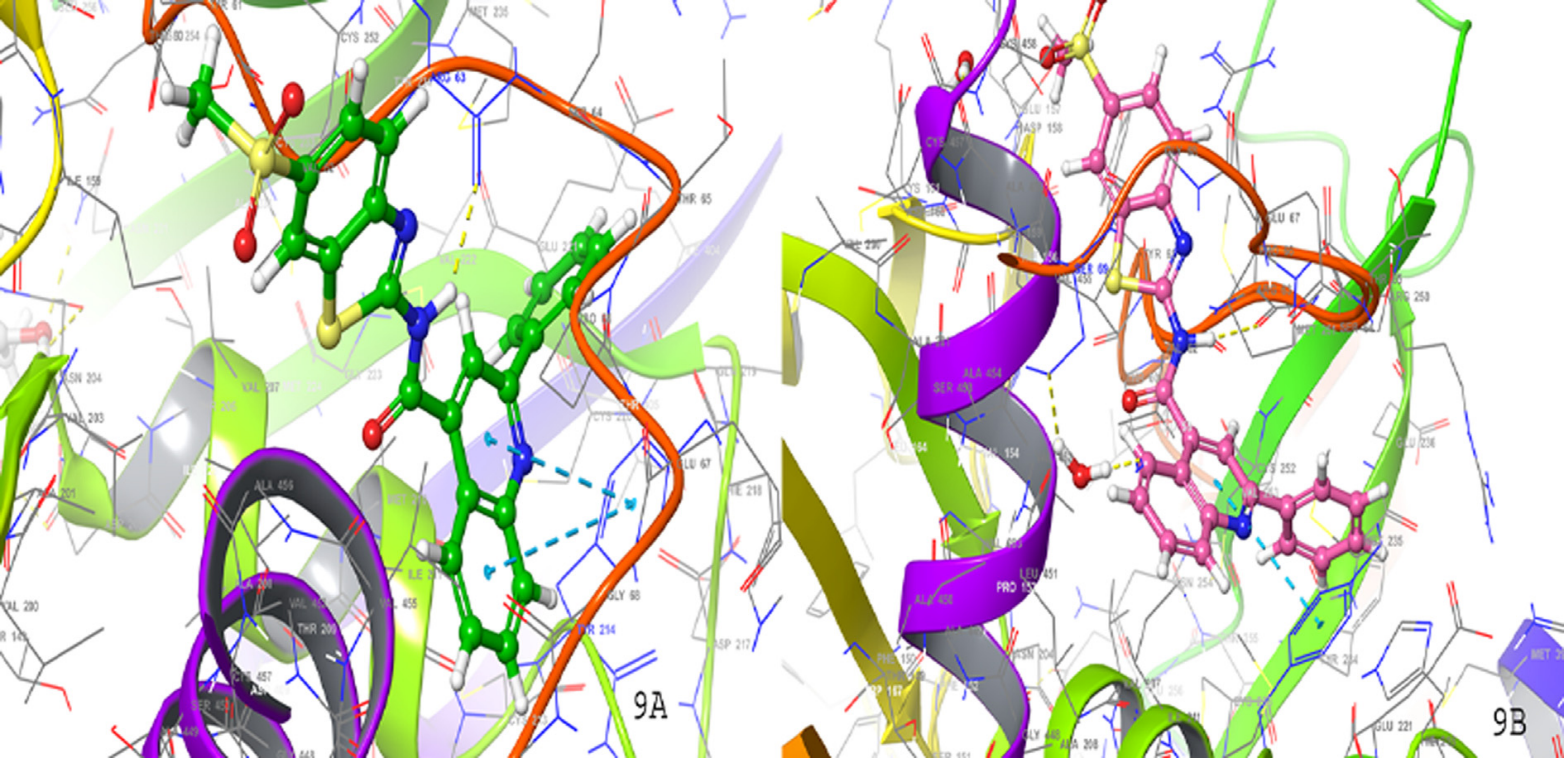
SP (9A) and XP (9B) ligand interaction diagram for higher scoring compound (ZINC08974524) screened through ZINC database.

The ZINC library was downloaded through the Swiss screening database. Total 3563 compounds were downloaded by ZINC database and further screened by different docking methodologies using Glide module. After applying Lipinski rule, the compounds were filtered through HTVS docking process. The top 50% of the compounds from this process were further taken for SP, and the top 20% compounds were finally taken for XP. Top hit, namely ZINC08974524 (Fig. 9) showed best docking score in SP (−11.17 kcal/mol), XP (−8.43 kcal/mol), and HTVS (−10.26 kcal/mol). The binding interactions of all the ZINC compounds were similar to crystal ligand interactions. The RMSD value was used as a parameter to check the binding pattern of different compounds from crystal ligand. These binding interactions of active compounds and ZINC derivatives displayed similar interaction as shown by crystal ligand of PDB ID-3A0I. The Zinc hit compounds may be used for the *in vivo* evaluation as GK-A. Furthermore, ZINC screening data suggested that the binding interactions were similar to the compounds taking for 3D QSAR study. So, these compounds may be designed further for the synthesis of potent compounds as GK-A against diabetes.

In ADME analysis, compounds showed solubility range from low to high values. The GI absorption was low for 16a and high for 19a compounds. The SwissTargetPrediction results exhibited that compounds have high target specificity for GK-A. All compounds showed good synthetic accessibility within the range of 2–4. These compounds may be useful for the generation of novel compounds as GK-A. The compounds screened through ZINC database showed good ADME properties **(**Table 9**)**. However, cytochrome profiling for ZINC hits shown inhibitory activities against CYP3A4, CYP2C19, and CYP2C9. All the compounds of the series and ZINC hits showed noncarcinogenic activities.

## 5 SAR optimization

**Fig. 10:**
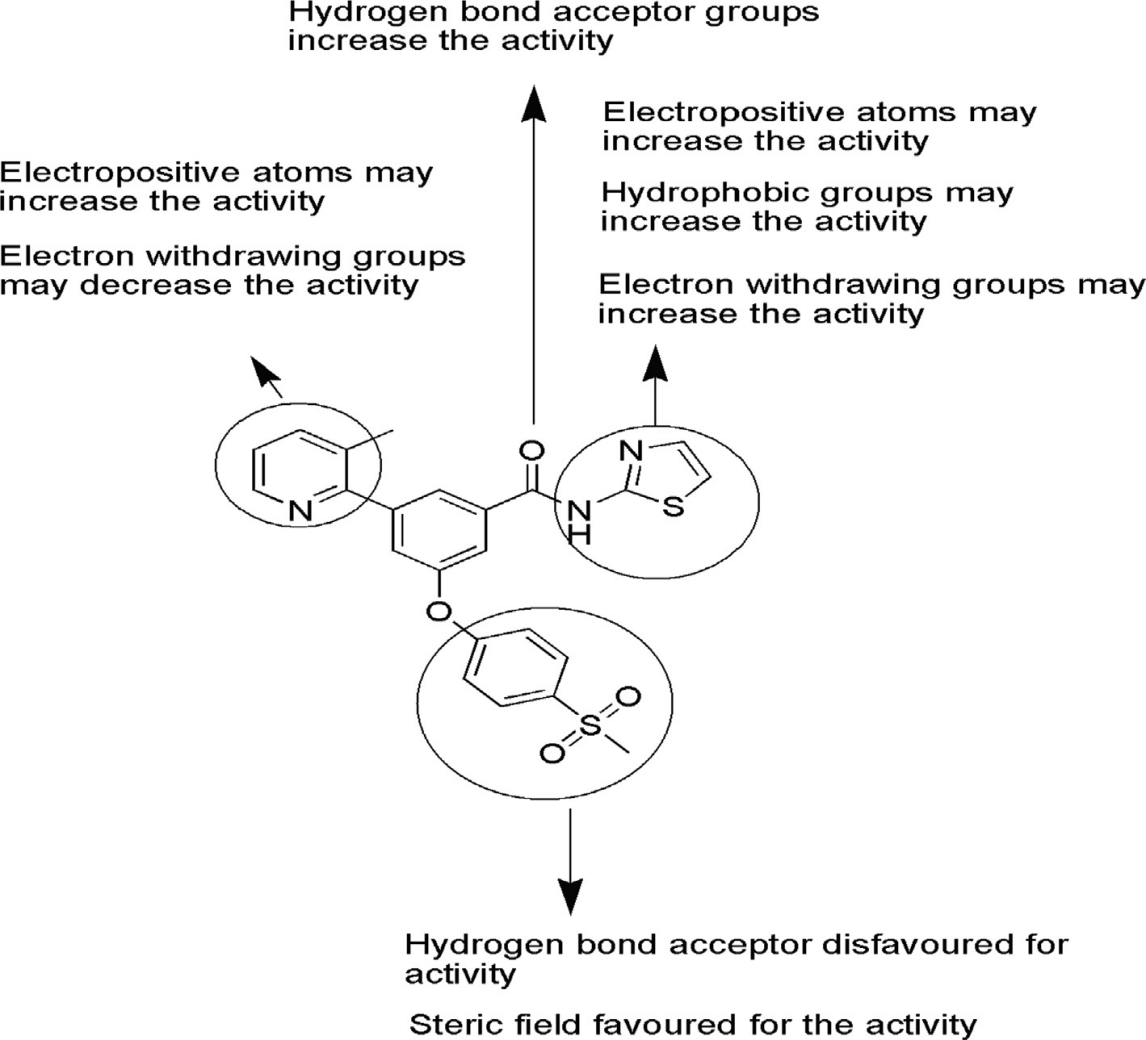
Substitutions on benzamide scaffold with different characteristic features for the development of future compounds as GK-A.

The pharmacophore model, 3D QSAR, virtual screening, and Zinc hit compounds may be used as a basis for the production of novel compounds as GK-A depicted in Fig. 10. The present SAR optimized by the 3D QSAR study revealed that the substitutions on benzamide scaffold with different characteristic features for the development of novel GK-A. The core moiety in the place of thiazole ring can be replaced by some electropositive atoms and hydrophobic groups such as long carbon chain and phenyl ring responsible for increase in activity. The oxygen atom of the benzamide group is more prominent for activity, or if it is replaced by less electronegative groups such as sulphur or nitrogen, then the activity may be diminished. The pyridine ring connected to the benzamide scaffold can be replaced by electropositive groups that results an increase in activity. These features can be used for the further development and synthesis of novel derivatives as GK-A.

## 6 Conclusion

The present manuscript revealed a computation study on benzamide derivatives processed in a sequence to produce potent GK-As, where ligands and ZINC hits showed no violation of the Lipinski rules. All screened compounds displayed good synthetic accessibility. Based on 3D-QSAR, pharmacophore development, SwissTargetPrediction, virtual screening, and molecular docking studies, we may design novel molecules with good ADME properties and low toxicity. ADRR_1 is determined as the best pharmacophore in the study. The 3D QSAR study showed the best statistical data by atom-based and field-based models, consecutively. The binding interactions of compounds showed important amino acids required for activity. Fig. 10 showed the importance of different substituents on core moiety which may help for the development of novel compounds as GK-A. Furthermore, the present study may be helpful for researchers as a guiding tool for the development of novel benzamide derivatives as GK-A.

## Declaration of Competing Interest

The author declare that there is no known competing financial interests or personal relationships that could have appeared to influence the work reported in this paper.
